# Gut Microbiome: The Interplay of an “Invisible Organ” with Herbal Medicine and Its Derived Compounds in Chronic Metabolic Disorders

**DOI:** 10.3390/ijerph192013076

**Published:** 2022-10-11

**Authors:** Dong-Woo Lim, Jing-Hua Wang

**Affiliations:** 1 Department of Diagnostics, College of Korean Medicine, Dongguk University, Dongguk-Ro 32, Goyang 10326, Korea; 2 Institute of Bioscience & Integrative Medicine, Daejeon University, 75, Daedeok-daero 176, Seo-gu, Daejeon 35235, Korea

**Keywords:** metabolic disorder, gut microbiota, herbal medicine, drug metabolism, drug–gut microbe interaction

## Abstract

Resembling a concealed “organ” in a holobiont, trillions of gut microbes play complex roles in the maintenance of homeostasis, including participating in drug metabolism. The conventional opinion is that most of any drug is metabolized by the host and that individual differences are principally due to host genetic factors. However, current evidence indicates that only about 60% of the individual differences in drug metabolism are attributable to host genetics. Although most common chemical drugs regulate the gut microbiota, the gut microbiota is also known to be involved in drug metabolism, like the host. Interestingly, many traditional herbal medicines and derived compounds are biotransformed by gut microbiota, manipulating the compounds’ effects. Accordingly, the gut microbiota and its specified metabolic pathways can be deemed a promising target for promoting drug efficacy and safety. However, the evidence regarding causality and the corresponding mechanisms concerning gut microbiota and drug metabolism remains insufficient, especially regarding drugs used to treat metabolic disorders. Therefore, the present review aims to comprehensively summarize the bidirectional roles of gut microbiota in the effects of herbal medicine in metabolic diseases to provide vital clues for guiding the clinical application of precision medicine and personalized drug development.

## 1. Introduction

Since our host origins, trillions of microbes have coexisted and coevolved with humans in the gastrointestinal tract [1]. In an innovative concept, the host and its commensal microbiomes are considered a “supraorganism” [2]. Because various microorganisms can be difficult to culture and because of the limitations of the technology for differentiation, investigations of the gut microbiota (GM) had progressed very slowly in the past [3]. However, recently, with the development of OMICs approaches, many scientists and physicians have established that ten times the cell number and one hundred times the genes exist in the GM compared to the human host itself [4,5]. Moreover, some researchers also estimated that the difference in the number of human cells and GM is not significant [6]. Although the numbers of commensal gut bacteria and their genes are debated by scholars [6], in recent decades, huge numbers of gut commensal bacteria with a tremendous number of genes have been proved to play a critical role in host metabolism, including drug metabolism [7]. Therefore, the studies concerning the relationship between GM-produced drug metabolites and host metabolism dysfunction are noteworthy.

In modern society, metabolic disorders (MetD) are common diseases often referred to as a new pandemic [8], with increasing prevalence [9]. MetD are heterogeneous diseases that occur when the normal metabolic process is disrupted due to abnormal chemical reactions [10]. These abnormal chemical reactions can lead to the maldistribution of macronutrients such as protein, fat, and carbohydrates [11]. Thus, at the physical level, weight loss or gain (in terms of the body mass index) is the primary sign of MetD; at the physiological level, high blood pressure is the primary sign of MetD; and, at the biochemical level, high triglyceride and high carbohydrate levels in the blood are the primary indicators of MetD [12,13,14,15]. These increase the risks of hyperlipidemia, hyperglycemia, and hypertension, resulting in obesity, diabetes, and cardiovascular diseases [16].

For the treatment of MetD, both synthetic and traditional medicines (herbal drugs/formulations) can be considered [17,18,19]. Each kind of medical system has its unique way of maintaining health. Generally, Western medicines are primarily metabolized in the liver via cytochrome P450 enzymes and impact host physiology [20,21]. In the past, it has been supposed that a single drug is appropriate for a single symptom for any individual. However, we still do not fully understand how a drug is metabolized in a particular individual for a particular disease. 

Decades earlier, it had already been established that every individual has a unique composition of intestinal bacteria, which can be recognized as commensal, opportunistic, and pathogenic [22]. The GM composition fluctuates due to multifactorial host conditions such as age, genetics, diet, drugs, and various environmental factors [23]. Many scientific findings have already revealed that the GM can directly contribute to MetD by increasing gut permeability and systemic low-grade inflammation [24]. Moreover, it is widely assumed that the host GM has a secondary impact on MetD by modulating the efficacy or availability of drugs taken by the host. 

To the best of our knowledge, drug metabolism comprises a sequence of complex processes regulated by host genetics, the GM composition, and environmental factors [25,26,27]. Current evidence indicates that only about 60% of the individual differences in drug metabolism are attributable to host genetics [28]. The GM fundamentally modulates drug metabolism through various enzymes, such as reductases, hydrolases, transferases, and lyases [29]. One ex vivo experiment showed that at least one GM species from 76 human gut commensal bacteria chemically modified approximately two-thirds of common clinical drugs [30]. Moreover, even a single species of the GM can metabolize 11 to 95 kinds of clinical drugs obtained from DrugBank (https://go.drugbank.com, accessed on 4 January 2018). Similarly, many herbal medicines and their derived compounds are biotransformed by GM, manipulating the drugs’ effects and safety [31]. 

Consequently, we present a comprehensive overview of advances regarding the GM and herbal medicines’ metabolism in MetD and the challenges at the frontiers of this rapidly accelerating field. The current review aims to summarize the outcomes of drug metabolism by the GM in metabolic diseases, which will help researchers to decide their directions of study. Meanwhile, it will provide a vital reference guiding the clinical application of precision medicine and personalized therapy for metabolic disorders. Ultimately, we hope the present overview can contribute to ameliorating the public health issue by widening the understanding of GM and their metabolism of natural drugs.

In the current study, the literature was searched through two well-known databases of biomedical literature, PubMed (www.ncbi.nlm.nih.gov/pubmed, accessed on 8 January 2022) and Google scholar (scholar.google.com), with the combinations of the following keywords: “herb”, “plant”, “herbal medicine”, “herbal drug”, “gut microbiota”, “gut microbiome”, “bioconversion”, “fermentation”, “metabolic diseases”, “metabolic syndrome”, “obesity”, “diabetes”, “NAFLD”, “NASH”, “fatty liver” and “hyperlipidemia”, whereas without time limitation. Eventually, the papers were selected by whether they contain microbial metabolites derived from herbal medicine and their natural compounds and relate to various metabolic diseases. 

## 2. The GM’s Interplay with Herbal Medicine, Altering Drugs’ Efficacy in Metabolic Disorders

Many studies have reported that the GM influences herbal drugs’ efficacy during microbial metabolism by changing pharmacokinetic processes [32]. As typical herbal-origin compounds, glycosides consist of one/several sugar(s) combined with an aglycone [33]. These phytochemicals are secondary plant metabolites and can be present along with phenols, alcohols, flavonoids, saponins, and anthraquinone [34]. However, the herb-derived glycosides are usually inactive due to their conjugated sugar moiety [35]; therefore, they are classified as prodrugs [36]. Nonactivated glycosides can be degraded/metabolized by the GM by their enzymes, producing bioactive aglycones [37]. In the process of microbial transformation, the properties of herbal medicine (HM) compounds have been shown to be greatly changed by general modifications into smaller, less polar, and more lipophilic molecules [38]. The above processes derived from the GM consist of many enzymatic reactions, such as the hydrolysis, oxidation, reduction, and esterification of the functional groups of compounds [39]. The GM-specific bioconversion processes of herbal compounds are highly differentiated into several stages and have distinct structural preferences in functional groups conducted cooperatively or independently [38]. 

The efficacy of herbal compounds can be modulated by changing their oral bioavailability [40]. In some cases, smaller molecules produced by digestion exhibit stronger efficacy than their parent molecules [31]. The GM also regulates the toxicity of HMs by metabolizing toxic substances [31]. The alteration of herbal toxicity by GM metabolism remains unclear and requires further investigation. Hence, we summarize how the GM modulates the efficacy of HMs used in the treatment of metabolic disorders. To readily comprehend the overview, we organized microbial metabolites of herb-derived compounds produced by gut microbiota in Table 1; we also arranged the impact of microbial metabolism on drug efficacy against metabolic diseases in Table 2. Meanwhile, the molecular and pharmacological properties of major compounds and their metabolites from herbs were listed in Table 3.

### 2.1. Gut Microbial Metabolism Produces Ginsenosides from Ginseng Radix, Exerting Bioactivity

The ginsenosides are a group of steroidal glycosides and triterpenes derived from ginseng that have pharmacological activity against diabetes, obesity, and other MetD [69]. The GM biotransformation process on ginseng saponins and its influence on host health have been extensively studied [70]. Previous findings revealed that the therapeutic potential of ginseng saponins largely depends on their bioconversion by the host GM, which can result in varying bioavailability, membrane permeability, and stability in the gastrointestinal tract [71]. The biological conversion of ginsenosides has been investigated in various studies, including ex vivo studies (anaerobic incubation with human fecal supernatants), in vivo studies (germ-free or antibiotic-treated animals, and gnotobiotic animals), and clinical trials. The 20(S)-protopanaxadiol-type ginsenosides (Rb1, Rb2, Rb3, Rc, and Rd) are mainly transformed into compound K, and Rh2 and 20(S)-protopanaxatriol-type ginsenosides (Re, Rg1, and Rg2) can also be converted into Rh1 and protopanaxatriol [70,72]. GM species, such as *Fusobacterium*, *Eubacterium*, and *Bifidobacterium* spp., predominantly biotransform the ginsenosides through β-glucosidase [41,42,44,45,46,47,70,73,74,75,76]. Among these bacterial metabolites, compound K, which is a hydrophobic and absorbable compound [73], has the most potent activity against numerous diseases, including various metabolic disorders [46]. 

### 2.2. Gut Microbial Metabolism Produces Active Compounds from Puerariae

Puerariae Radix, enriched with isoflavone glucosidases, has a long history of use in east Asia, possessing therapeutic effects on obesity, dyslipidemia, and insulin signaling [77,78]. The typical compounds in Puerariae Radix include puerarin, daidzin, and daidzein [79]. Daidzin and puerarin are metabolized into daidzein and, further, into equol, which is promising for estrogenic activities [80]. It was demonstrated that daidzein shows higher intestinal absorbability than daidzin in the Caco-2 cell model, implying the importance of bacterial hydrolysis in absorption [81]. Another in vitro study revealed that daidzin and puerarin were transformed into daidzein by human fecal bacteria, such as *Eubacterium* A-44, and the metabolite daidzein displayed effectively increased estrogenic activity [51]. Other flavonoids are found in Pueraria flos, including kakkalide and tectoridin, which also have estrogenic effects similar to those of equol [50]. In this case, kakkalide and tectoridin are mainly metabolized into irisolidone and tectorigenin by the human and rat gut bacterium *Bifidobacterium* K-110 via β-D-xylosidase, and they exert stronger activity than their corresponding precursors [50,52]. 

### 2.3. Gut Microbial Metabolism of Compounds from Coptidis Rhizoma Improves Their Absorption Rate

Flavone glycosides and berberine are the main active compounds from Coptis Chinensis, which exerts notable effects on type 2 diabetes (T2DM) and T2DM-related complications, including hyperlipidemia, heart disease, and retinopathy [82]. Although it is an essential compound from Coptidis rhizoma with many properties, berberine has extremely low bioavailability (<1%) [83], and its absorption is largely attributed to the activity of GM [84]. Berberine can be metabolized by the GM into dihydroberberine, berberrubine, demethyleneberberine, jatrorrhizine, and oxyberberine [85]. The biotransformation of berberine into the reduced form, dihydroberberine, is achieved by *Enterobacter cloacae* and *Enterococcus faecalis* by nitroreductase, improving its absorption rate [86]. Once absorbed, dihydroberberine is reverted to berberine in the host’s intestinal epithelial tissue and dispersed to organs, where it exerts its pharmacological activities [55]. Another metabolite, oxyberberine, is metabolized by the intestinal microbiota, showing greater effects than berberine [53]. 

### 2.4. Gut Microbial Bioconversion of Compounds from Scutellaria Radix Improves Their Absorption Rate

The root of *Scutellaria baicalensis* and its major compound, baicalin, have been used to treat metabolic diseases, including obesity, hyperlipidemia, metabolic syndrome, and diabetes [87]. Baicalin is hydrolyzed into its aglycone, baicalein, by β-glucuronidase from E. coli [57] and is thereby easily absorbed in the intestine [88]. Absorbed baicalein can be reconjugated into baicalin by UDP-glucuronosyltransferase in the host’s liver and intestine and exert beneficial activities [56,84]. An in vivo study using a bile-duct-ligated rat model suggested that baicalin is converted to baicalein by the GM generating β-glucuronidase, and that the absorption of baicalein is preferable to that of baicalin in the gastrointestinal tract [89]. Wogonin is another key component of *Scutellaria baicalensis*. As an aglycone derived from wogonoside, it has a beneficial effect on glucose and lipid metabolism [90]. A rat study demonstrated the fundamental role of the GM in the absorption of compounds from *Scutellaria baicalensis*, in which antibiotic pretreatment inhibited the absorption of wogonoside and baicalin and its metabolites [91]. Intestinal bacteria of the *Lactobacillus* spp. and their glucuronidase enzymes are reported to be involved in these enzymatic reactions [58,59], which also increases the bioavailability of compounds.

**Table 2:** Gut microbial metabolites derived from herbal compounds and their Impact on metabolic diseases.

Herb Name	Microbial Metabolites	Treatment of Diseases	Study Design (In Vitro/In Vivo/Clinical Study)	Impact of Drug Efficacy	Ref.
Ginseng Radix	Compound K	Diabetes	In vivo (SD rats) In vitro (Caco-2 cell permeability)	Increased absorption	[73]
	Compound K Ginsenoside Rh1	NAFLD	In vivo (HFD-fed SD rats) In vitro (HSC-T6 cell)	Increased activity	[92]
	Compound K	Diabetes	In vivo (STZ and HFD-fed ICR mice)	Increased activity	[93]
Puerariae Radix and Puerariae Flos	Irisolidone Tectorigenin	Estrogenic effect	In vitro (human fecal incubation, MCF-7 cells)	Increased activity (c-fos and pS2 gene)	[50]
	Daidzein	Not indicated	In vitro (Caco-2 permeability) In vivo (hydrolyzation by rat microvilli)	Increased absorption	[81]
	Daidzein	Estrogenic effect	In vitro (human fecal incubation, MCF-7 cells)	Increased activity	[51]
	Equol	NAFLD	In vivo (HFD-fed mice)	Increased activity Changed bioactivity	[94]
Coptidis Rhizoma	Oxyberberine	Colitis	In vivo (DSS-induced colitis Balb/C mice)	Increased activity	[53]
	Dihydroberberine	Diabetes	In vivo (KK-Ay mice)	Increased absorption	[55]
	Berberrubine	Hypercholesterolemia	Clinical study (*n* = 12, moderate hypercholesterolemia)	Increased activity	[95]
Scutellaria Radix	Baicalein	Not intended	In vivo (antibiotic-treated SD rats)	Increased absorption	[96]
	Baicalein	Not intended	In vivo (germ-free Wistar rats)	Increased absorption	[56]
	Baicalein	Not intended	In vivo (bile-duct-ligated Wistar rats	Increased absorption	[89]
	Wogonin	Not intended	In vivo (antibiotic-treated SD rats)	Increased absorption	[91]
Curcumae Radix	Tetrahydrocurcumin	Diabetes	In vivo (STZ-induced diabetic rats)	Increased activity	[97]
	Tetrahydrocurcumin	Lipid accumulation	In vitro (THP-1 cells)	Decreased activity	[98]
Mori folium, Bupleurum Radix, Houttuyniae Herba	Quercetin	Platelet activity	In vitro	Increased activity	[65]
	Quercetin	Insulin resistance	In vitro (TNF-α-treated C2C12 cells)	Increased activity	[99]
Glycyrrhizae Radix	Glycyrrhetic acid	Not indicated	In vivo (SD rats, Wistar germ-free rats)	Increased bioavailability	[100]
	18β-Glycyrrhetinic acid	Obesity	In vitro (3T3-L1) In vivo (HFD-fed C57/BL6 mice)	Not indicated	[101]
	18β-Glycyrrhetinic acid	NASH	In vivo (MCD; C57/BL6 mice)	Increased bioactivity	[90]

SD, Sprague Dawley; STZ, streptozotocin; HFD, high-fat diet; HSC, hepatic stellate cell; NAFLD, non-alcoholic fatty liver disease; DSS, dextran sulfate sodium; TNF, tumor necrosis factor; NASH, non-alcoholic steatohepatitis; MCD, methionine- and choline-deficient diet.

### 2.5. Gut Microbial Metabolism of Curcumin from Curcumae Radix Increases Its Bioavailability 

Curcumae Radix contains curcumin, a phenolic pigment insoluble in water, which shows pharmacological activities against metabolic diseases, including obesity, diabetes, and hepatic steatosis [102,103]. As a polyphenol, curcumin has low bioavailability as demonstrated by its in vivo pharmacokinetic data [104]. The main reasons for the low bioavailability of curcumin are its poor absorption, instability, rapid metabolism, and rapid excretion [105]. However, curcumin can be metabolized by the human gut bacteria *Blautia* sp. MRG-PMF1 into demethylcurcumin and bisdemethylcurcumin [62]. Additionally, an in vitro fermentation study reported that three bacteria, including *Escherichia fergusonii and Escherichia coli* DH10B, metabolized curcumin via two-step reduction into dihydrocurcumin as an intermediate, followed by tetrahydrocurcumin and ferulic acid as final products [60]. The debate over any difference in biological activity between the parent compound (curcumin) and its major metabolite (tetrahydrocurcumin) is ongoing; however, it seems that they possess differential activity with distinct target molecules [104]. 

### 2.6. Gut Microbial Bioconversion of Quercitrin from Several Herbs into Quercetin Increases Its Bioavailability 

Quercetin and its glycoside form, quercitrin (quercetin 3-rhamnoside), are the most common flavonoids found in nature [106]. These compounds are distributed in some common traditional medicinal herbs and foods, like Mori folium, Bupleurum Radix, and Houttuyniae Herba [65,107,108]. Like other flavonoids, quercetin glycosides are not bioavailable due to their structures [84]; however, intestinal microbiota including *Bacillus subtilis* and *Fusobacterium* K-60 can metabolize quercitrin to produce quercetin through dioxygenase or α-L-rhamnosidase [63,109]. Among the aglycones, quercetin possesses ubiquitous effects of hypoglycemic, hypolipidemic, and hypotensive and anti-obesity with multifaceted mechanisms [110]. Meanwhile, the low bioavailability of quercitrin also affects its delivery into farther regions of the intestine, where it can be decomposed to quercetin, the active aglycone [111].

### 2.7. Glycyrrhizin from Glycyrrhizae Radix Requires Bacterial Transformation to Be Absorbed in the Intestine 

Glycyrrhizin is a triterpenoid saponin derived from Glycyrrhizae Radix (licorice) that is used for its various clinical indications, including nonalcoholic fatty liver disease, gastric disorders, and metabolic disorders [112,113]. Extracted licorice contains glycyrrhizin and its aglycone, glycyrrhetic acid, as bioactive compounds. An in vivo study showed that the administration route of glycyrrhizin is critical for its bioavailability; that bioavailability under oral administration was approximately 1% due to its poor absorption in the intestine [114]. The bioconversion of glycyrrhizin to an active form, 18β-glycyrrhetinic acid or glycyrrhetic acid, occurs in the presence of β-D-glucuronidase from *Eubacterium*, *Ruminococcus*, and other species of the intestinal microbiota [115].

**Table 3:** The molecular and pharmacological properties of major compounds and their metabolites from herbs, described in the main text.

Herbal Medicine	Raw Compound	Properties of Raw Compound	Properties of Metabolite	Metabolite
Ginseng Radix	Ginsenoside Rb1	PubChem CID		Ginsenoside Rd
	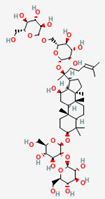	9898279	11679800	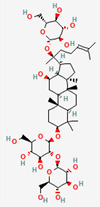
		Molecular Weight		
		1109.3	963.30	
		Bioavailability Score		
		0.17	0.17	
		GI Absorption		
		Low	Low	
		Lipinski’s Criteria		
		No (3 violations) MW > 500, NorO > 10, NHorOH > 5	No (3 violations) MW > 500, NorO > 10, NHorOH > 5	
	Ginsenoside Rc	PubChem CID		Compound K
	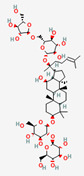	12855889	5481990	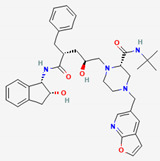
		Molecular Weight		
		1079.3	653.8	
		Bioavailability Score		
		0.17	0.55	
		GI Absorption		
		Low	High	
		Lipinski’s Criteria		
		No (3 violations) MW > 500, NorO > 10, NHorOH > 5	Yes (1 violation) MW > 500	
Puerariae Radix and Puerariae Flos	Daidzin	PubChem CID		Daidzein
	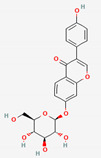	107971	5281708	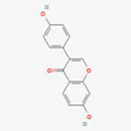
		Molecular Weight		
		416.41	254.24	
		Bioavailability Score		
		0.55	0.55	
		GI Absorption		
		Low	High	
		Lipinski’s Criteria		
		Yes (0 violations)	Yes (0 violations)	
Coptidis Rhizoma	Berberine	PubChem CID		Oxyberberine
	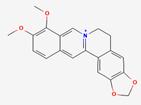	2353	11066	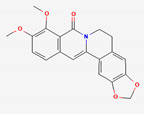
		Molecular Weight		
		336.4	351.4	
		Bioavailability Score		
		0.55	0.55	
		GI Absorption		
		High	High	
		Lipinski’s Criteria		
		Yes (0 violations)	Yes (0 violations)	
Scutellaria Radix	Baicalin	PubChem CID		Baicalein
	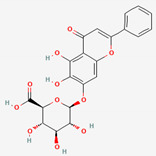	64982	5281605	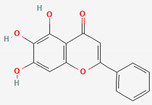
		Molecular Weight		
		446.4	270.24	
		Bioavailability Score		
		0.11	0.55	
		GI Absorption		
		Low	High	
		Lipinski’s Criteria		
		No (2 violations) NorO > 10, NHorOH > 5	Yes (0 violations)	
Curcumae Radix	Curcumin	PubChem CID		Dihydrocurcumin
	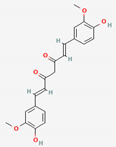	969516	10429233	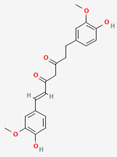
		Molecular Weight		
		368.4	370.4	
		Bioavailability Score		
		0.55	0.55	
		GI Absorption		
		High	High	
		Lipinski’s Criteria		
		Yes (0 violations)	Yes (0 violations)	
Mori folium/ Bupleurum Radix/Houttuyniae Herba	Quercitrin	PubChem CID		Quercetin
	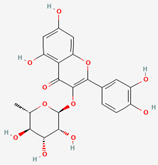	5280459	5280343	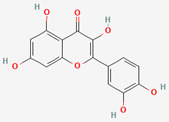
		Molecular Weight		
		448.4	302.23	
		Bioavailability Score		
		0.17	0.55	
		GI Absorption		
		Low	High	
		Lipinski’s Criteria		
		No (2 violations) NorO > 10, NHorOH > 5	Yes (0 violations)	
Glycyrrhizae Radix	Glycyrrhizin (Glycyrrhizic Acid)	PubChem CID		18-β-Glycyrrhetinic Acid (Glycyrrhetic Acid)
	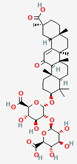	14982	10114	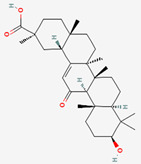
		Molecular Weight		
		822.9	470.7	
		Bioavailability Score		
		0.11	0.85	
		GI Absorption		
		Low	High	
		Lipinski’s Criteria		
		No (3 violations) MW > 500, NorO > 10, NHorOH > 5	Yes (1 violation) MLOGP > 4.15	

Data were obtained from the PubChem (https://pubchem.ncbi.nlm.nih.gov/, accessed on 7 August 2022) and SwissADME (http://www.swissadme.ch/, accessed on 7 August 2022) online databases.

## 3. Current Status and Future Perspectives 

Understanding the variability of the GM and host digestion of drugs is necessary for precision medicine [40]. As presented herein, natural drugs are metabolized by the host GM through complex mechanisms. Based on these findings, some studies have started to interpret the differential efficacies of herbal drugs between individuals, using the different gut microbial compositions. 

For instance, a study comparing two groups of Korean subjects with distinct capabilities for metabolizing compound K showed a marked difference in the compositions of their GM, which explained the inconsistency in the drug potency of Panax ginseng between individuals [116]. Another case is the Rhei Radix medicine used in postoperative patients; these patients are frequently administered antibiotics, which prevents the prodrug from being properly metabolized by the GM, and purgative efficacy was not observed in many cases [117]. Another consideration is the fact that the diversity of the GM varied across ethnicities, which could also influence the efficacy of natural drugs [118]. 

As mentioned above, a series of evidence indicated the undeniable impacts of individual GM on personal health for clinicians. Therefore, it is necessary to establish integrated databases containing herbal compounds and gut microbial metabolites according to the representative types of human microbial communities. However, current studies describing the impacts of microbial conversions of natural drugs on their efficacies are relatively scarce among the studies on drugs and are fragmented by their scope. For instance, most articles focus on the microbial conversion process itself, not exploring the differences in efficacy between the metabolites and parent compounds. Other researchers have only focused on the outcomes of microbial bioconversion, without exploring the bacteria or enzymes involved. Therefore, an integrated natural compound library should cover intact natural components, microbial metabolites, enzymes involved in the process, and predicted consequences for the oral bioavailability or bioactivities, to enable a better understanding and prediction of the impacts of natural products or herbal medicinal treatments on certain diseases. On the other hand, this also requires a metagenomic database of GM in various populations. Fortunately, the outcome of 845 intestinal microbial metagenome data analyses in three Asian countries was recently published (Korea, Japan, and India) [119].

However, the herbal drug–microbiota interaction is reciprocal, not unilateral. As many studies have revealed, herbs exert profound effects on the GM community, sometimes via bactericidal or prebiotic effects. Berberine has been reported to modulate the GM in rats with obesity induced by a high-fat diet [120], and this compound is known to exert antibiotic effects, especially on Gram-negative bacteria. In addition, the antidiabetic effects of baicalein are associated with modulation of the GM [121], and baicalein is also known to restrict the growth of harmful bacterial strains. On the contrary, herbal polysaccharides and glycosides usually possess prebiotic effects, providing carbohydrates as nutrients [40]. As a result, it has been demonstrated that modulation of the GM to ameliorate metabolic disturbances may now be a feasible strategy [122]. Therefore, the Impact of herbal drugs/prescriptions on the commensal gut bacterial community should also be considered, to optimize the use of natural drugs.

Only a small proportion of the interactions between natural drugs and GM have been elucidated, considering the huge contribution of bacterial metabolism in digestion [123,124]. A recent study suggested a novel solution: adopting machine learning to predict drugs’ metabolism by GM [125]. Although the model used in the study predicted the depletion of drugs by gut microbial metabolism and did not suggest any consequent metabolites, it is worth exploring the possibilities of computational analysis in this field. So far, it is still challenging to fully clarify how gut microbial metabolism benefits treating metabolic disease, even with the enhanced bioavailability of drugs. Herein, the selected publications revealed an increasing tendency in the recent decade; however, only 2% of human studies reflected the status of severe deficiency regarding herb–drug metabolism and gut microbiota, especially in metabolic dysfunction (Figure S1). Thus, the scientific evidence is still inadequate, especially from human trials. We anticipate that the complex interaction between GM and herbal medicines and the aftermath of microbial metabolism will be investigated clearly through more and more animal and clinical studies.

## 4. Conclusion 

Overall, the present review explored the roles of the GM in the metabolism of herbal compounds to provide a vital reference for guiding clinical applications and further research. This review also provides valuable clues to assist in the application of clinical drugs in precision medicine and should contribute to personalized drug development for metabolic diseases. For policymakers, good pharmacovigilance needs to consider the host commensal microbiota to guarantee public medication safety and effectiveness, especially for herbal medicine.

## Figures and Tables

**Table 1:** Herbal compounds and their microbial metabolites formed by host GM.

Herbal Medicine	Compound	Related Microbiota	Microbial Metabolites	Mechanisms	Ref.
Ginseng Radix	Ginsenoside Rb1	*Bifidobacterium longum* H-1	Ginsenoside Rd Compound K	β-D-glucosidase	[41]
	Ginsenoside Rb1	*Fusobacterium* K-60	Compound K	β-Glucosidase	[42]
	Ginsenosides Ra1 and Ra2	*Bifidobacterium breve* K-110	Ginsenosides Rb2, Rc	β-D-Xylosidase	[43]
	Ginsenoside Rb1	*Microbacterium esteraromaticum*	Ginsenoside Rd Ginsenoside 20(S)-Rg3	β-Glucosidase	[44]
	Ginsenoside Rb1	*Eubacterium* sp. A-44	Ginsenoside Rd Ginsenoside F2 Compound K	β-D-glucosidase	[45]
	Ginsenoside Rc	*Bifidobacterium* K-103 *Eubacterium* A-44	Ginsenoside Rd (intermediate) Compound K	Hydrolysis	[46]
		*Bacteriodes* HJ-15 *Bifidobacterium* K-506	Ginsenoside Mb (intermediate) Compound K	Hydrolysis	
	Ginsenoside Rb1	*Prevotella oris*	20-O-/J-o-glucopyranosyl-20(S)-protopanaxadiol	β-Glucosidase hydrolysis	[47]
Puerariae Radix And Puerariae Flos	Puerarin	*Dorea longicatena* PUE	Daidzein	Deglycosylation	[48]
	Daidzein	*Slackia isoflavoniconvertens.*	Equol	Not identified	[49]
	Kakkalide Tectoridin	*Bifidobacterium breve* K-110	Irisolidone tectorigenin	β-D-Xylosidase	[50]
	Puerarin Daidzin	*Bacteroides sterocoris* HJ-15 *Bifidobacterium longum* H-1 *Eubacterium rectale* A-44 *Streptococcus faecium* S-9	Daidzein	Hydrolysis	[51]
	Kakkalide Irisolidone	Not identified	Irisolidone Biochanin A	Hydrolysis Dehydroxylation Demethoxylation Demethylation Hydroxylation Decarbonylation Reduction	[52]
Coptidis Rhizoma	Berberine	*Escherichia coli* *Streptococcus faecalis* *Lactobacillus acidophilus*	Oxyberberine	Oxidation	[53]
	Berberine	Not identified	Thalifendine Berberrubine Jatrorrhizine	Not identified	[54]
	Berberine	*Enterobacter cloacae* *Enterococcus faecium*	Dihydroberberine	Nitroreductase	[55]
Scutellaria Radix	Baicalin	Not identified	Baicalein	Not identified	[56]
	Baicalin	*Escherichia coli*	Baicalein	Beta-D-glucuronidase	[57]
	Baicalin Wogonoside	*Lactobacillus delbrueckii* Rh2	Baicalein Wogonin	β-glucuronidase	[58]
	Baicalin Wogonoside	*Lactobacillus brevis* RO1	Baicalein Wogonin	β-glucuronidase	[59]
Curcumae Radix	Curcumin Demethoxycurcumin Bis-demethoxycurcumin	*Escherichia fergusonii* *Escherichia coli* ATCC 8739 *Escherichia coli* DH10B	Dihydrocurcumin Tetrahydrocurcumin Ferulic acid	Reduction (CurA)	[60]
	Curcumin	*E. Coli* strain DH10B	Dihydrocurcumin Tetrahydrocurcumin	Reduction (CurA)	[61]
	Curcumin (1) Demethoxycurcumin (2) Bisdemethoxycurcumin (3)	*Blautia* sp. MRG-PMF1	Dimethylcurcumin (from 1) Bisdemethylcurcumin (from 1) Demethyldemethoxycurcumin (from 2)	Reduction	[62]
Mori folium/ Bupleurum Radix/ Houttuyniae Herba	Quercitrin	*Bacillus subtilis*	Quercetin	Dioxygenase (C-ring cleavage)	[63]
	Quercitrin	*Fusobacterium* K-60	Quercetin	Hydrolysis (α-L-Rhamnosidase)	[64]
	Quercitrin	*Fusobacterium* K-60	Quercetin 3,4-Dihydroxyphenylacetic acid 4-Hydroxylphenylacetic acid	Not identified	[65]
Glycyrrhizae Radix	Glycyrrhizin	*Eubacterium* sp. GLH	18β-Glycyrrhetinic acid monoglucuronide 18β-Glycyrrhetinic acid	Deglycosylation	[66]
	Glycyrrhizin	Not indicated (human feces sample)	18β-Glycyrrhetic acid	b-D-glucuronidases	[67]
	Glycyrrhizin	*Ruminococcus* sp. PO1-3	18β-Glycyrrhetic acid 3-Oxo-glycyrrhetic acid	b-D-glucuronidases 3β-Hydroxysteroid dehydrogenase	[68]

## Data Availability

Not applicable.

## Supplementary Materials

The following are available online at https://www.mdpi.com/article/10.3390/ijerph192013076/s1. Figure S1: general information of selected papers in the present review.
