# Supplementary material for: Gut Microbiome: The Interplay of an “Invisible Organ” with Herbal Medicine and Its Derived Compounds in Chronic Metabolic Disorders
Source: Int J Environ Res Public Health. 2022 Oct 11;19(20):13076. doi: 10.3390/ijerph192013076 (PMC9603471; doi:10.3390/ijerph192013076)
Supplement: Supplementary file 1 [file ijerph-19-13076-s001.zip › ijerph-1898214-supplementary.pdf]

Figure S1. General information of selected papers in the present review.

A

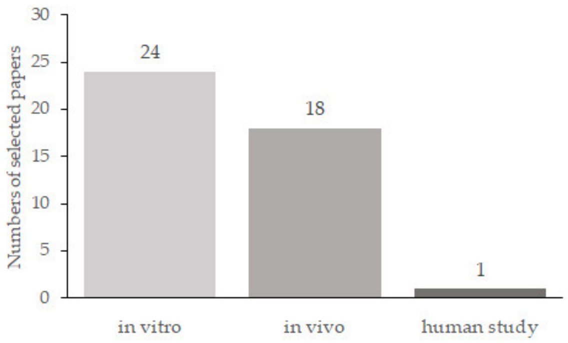

B

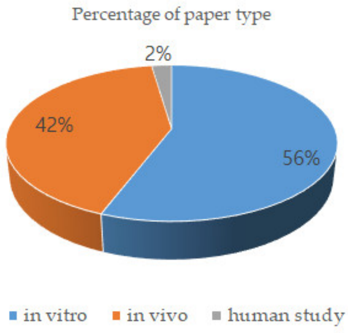

C

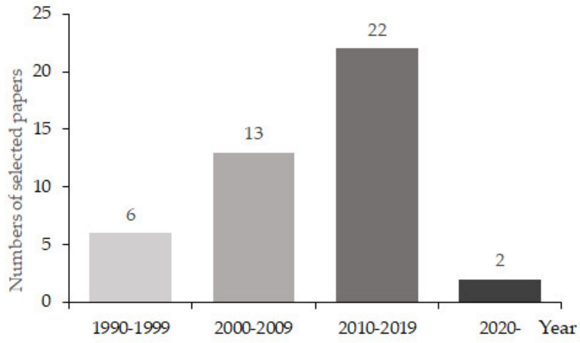

A) Numbers of selected papers based on the research types

B) Percentages of selected papers based on the research types

C) Numbers of selected papers based on the decade
